# Differential pathways from childhood maltreatment types to non-suicidal self-injury in adolescents with depressive disorders: a serial mediation model

**DOI:** 10.3389/fpsyt.2026.1960956

**Published:** 2026-09-30

**Authors:** Xiaorun Zhu, Ting Wang, Chaoyun Zhou, Meihui Gao

**Affiliations:** Department of Psychology, Wenzhou People’s Hospital, The Wenzhou Third Clinical Institute Affiliated To Wenzhou Medical University, Wenzhou, Zhejiang, China

**Keywords:** adolescents, childhood maltreatment, depressive disorders, depressive symptoms, Emotion dysregulation, Non-suicidal self-injury

## Abstract

**Background:**

Non-suicidal self-injury (NSSI) is prevalent among adolescents with depressive disorders, with childhood maltreatment (CM) as a key risk factor. However, how distinct CM types differentially influence NSSI risk in this population remains unclear. Moreover, emotion dysregulation and depressive symptoms have rarely been tested together as serial mediators within a single model. This study examined the serial mediating effects of emotion dysregulation and depressive symptoms linking five CM types to NSSI and compared their distinct pathways.

**Methods:**

A total of 426 adolescents with depressive disorders (218 males, 208 females; mean age = 14.74 years, SD = 1.93) were enrolled. CM, NSSI, emotion dysregulation, and depressive symptoms were assessed using the Childhood Trauma Questionnaire-Short Form, Functional Assessment of Self-Mutilation, the Difficulties in Emotion Regulation Scale-16, and the depression subscale of the Symptom Checklist-90, respectively. Serial mediation was assessed via structural equation modeling using the MLR estimator, with indirect effects tested via robust 95% confidence intervals (delta method).

**Results:**

Emotional abuse (β = 0.157) exerted direct effects on NSSI, as did physical abuse (β = 0.179), while neglect types did not. Indirect pathways varied by maltreatment type: emotional abuse operated through emotion dysregulation alone (β = 0.087), depressive symptoms alone (β = 0.026), and the serial pathway (β = 0.036). Both emotional and physical neglect demonstrated indirect effects through emotion dysregulation (β = 0.097 and 0.091, respectively) and through the serial pathway (β = 0.041 and 0.038, respectively), but physical neglect did not operate through depressive symptoms alone. Formal contrasts showed no differences in total indirect effects among emotional abuse, emotional neglect, and physical neglect, whereas physical abuse and sexual abuse had significantly weaker total indirect effects. Sexual abuse showed no significant effects.

**Conclusion:**

Five CM types influence NSSI risk through differential pathways in adolescents with depressive disorders. Emotion dysregulation served as a shared mediating mechanism for emotional abuse and neglect, but not for physical abuse or sexual abuse. Emotion regulation training may be beneficial for reducing NSSI among depressed adolescents exposed to emotional abuse or neglect.

## 1 Introduction

Non-suicidal self-injury (NSSI) describes intentional, direct harm to bodily tissue in the absence of suicidal intent (1). In China, 24.7% of students aged 5-25 report lifetime NSSI and this figure rises to 62.6% among adolescents with mood disorders (2, 3). Adolescence constitutes a peak period for NSSI, with onset typically peaking between 14 and 15 years of age (4). NSSI elevates the risk of subsequent suicidal behavior (5) and substance abuse (6), in addition to generating a considerable burden for families and healthcare systems. Nevertheless, while persistent depressive symptoms constitute a key risk factor sustaining NSSI behaviors (7), individual differences in NSSI among depressed adolescents remain insufficiently clarified. For NSSI prevention and intervention, elucidating the mechanisms driving such disparities is essential for identifying high-risk subgroups and developing strategies.

Childhood maltreatment (CM) represents a prevalent form of early adversity, typically classified as five types (8). These include emotional neglect (EN), physical neglect (PN), emotional abuse (EA), physical abuse (PA), and sexual abuse (SA). Within the integrated theoretical model of self-injury, childhood maltreatment represents one distal risk factor that may heighten vulnerability to interpersonal difficulties, thereby elevating the risk of self-injury (9). The developmental model proposes that childhood maltreatment impairs critical developmental competencies, with NSSI potentially emerging as a maladaptive coping mechanism for stress and affect regulation (10).

Robust evidence confirms the connection between CM and NSSI, yet the magnitude of this relationship differs considerably across maltreatment types. Studies on emotional abuse consistently rank it as the strongest predictor of NSSI across maltreatment types (OR = 3.03) (11), a pattern that has been replicated in clinical depressed adolescents (12). However, some studies have shown that adjusting for overlapping maltreatment types attenuates or even eliminates this independent predictive effect (13), suggesting that unadjusted associations may partially reflect shared variance with other forms of maltreatment (14).

Physical abuse is positively associated with NSSI, although its effect sizes are weaker than those of emotional abuse (11, 15). This relationship has also been observed among depressed adolescents (16). However, findings are not entirely consistent. In an inpatient youth sample, physical abuse showed no direct association when other maltreatment types were accounted for (17). Whether physical abuse directly or indirectly predicts NSSI in this population remains unclear.

Unlike the convergent evidence for emotional and physical abuse, research on sexual abuse presents marked heterogeneity. Some studies report significant associations (11), whereas others find the association substantially reduced or non-significant after controlling for psychiatric risk factors (18) or when all maltreatment types are considered simultaneously (19). This inconsistency extends to clinical samples: sexual abuse significantly predicted NSSI (OR = 1.60) (13), whereas no independent effect was reported when other maltreatment types were accounted for (17). Whether sexual abuse independently contributes to NSSI in clinical depressed adolescents, and through what mechanisms, remains to be determined.

Notably, studies focusing on neglect-NSSI association remain relatively limited. One investigation has found neglect significantly predicts NSSI (15), although a large-scale meta-analysis found that this association does not hold specifically for emotional neglect (11). Even so, existing work indicates that both emotional neglect (20) and physical neglect (21) exert effects on NSSI exclusively through indirect pathways rather than direct routes. These inconsistent findings likely stem from cross-study heterogeneity in maltreatment definitions, measurement tools, and culturally shaped assessment practices (15). Furthermore, most available evidence is derived from community samples, and it remains unknown whether the neglect–NSSI pathway generalizes to clinical adolescents with depression.

Taken together, maltreatment types differ not only in their associations with NSSI but also in the pathways through which they confer risk. These differences partly reflect etiological heterogeneity and methodological variation across studies, including sample characteristics (16), measurement approaches (11), and differences between clinical and community samples. However, a persistent limitation in this literature is that co-occurring CM types contribute to NSSI risk remains insufficiently understood (22). Consequently, whether these patterns extend to clinical depressed adolescents and through what mechanisms remains an open question.

One pathway linking CM to NSSI risk is through depressive symptoms, although the strength of this association varies across maltreatment types. In a youth-focused meta-analysis, the strongest link to depression was found for EA (OR = 4.25), followed by EN (OR = 3.09), while SA demonstrates the weakest association (OR = 1.85) (23). In contrast, evidence derived from clinically diagnosed major depressive disorder samples reveals divergent patterns: emotional neglect was the strongest predictor (OR = 3.38), whereas emotional and physical abuse did not reach statistical significance (24). Such discrepancies indicate that the maltreatment type and depression association is jointly shaped by maltreatment type, developmental timing, and sample characteristics.

Furthermore, depression and NSSI are closely interrelated (25), and the risk of NSSI engagement is substantially elevated among depressed individuals (26). The experiential avoidance model provides a framework for understanding NSSI, which is primarily perpetuated through negative reinforcement by escaping or avoiding unwanted emotional experiences (27). As a core form of negative affective experience, depression mediates the impact of CM on NSSI (28). However, it remains uncertain how different maltreatment types influence NSSI via depression. The mediating effect of depressive symptoms has been reported to vary across CM types, ranging from 45.5% for physical abuse to 86.5% for neglect (29). The mediating role of depression has been supported for emotional abuse and NSSI but not emotional neglect (21), whereas others have found that neglect operates through a fully mediated pathway via depression (30). These discrepancies highlight the need for further investigation into the mediating role of depression, particularly for neglect, in the pathway from maltreatment to NSSI.

Emotion dysregulation (ED) refers to impairments in the awareness, understanding, or regulation of emotions, which may disrupt adaptive functioning (31) and lead to adverse outcomes including depression (32) and self-injurious behaviors (33). The emotion regulation model of NSSI proposes that self-injury serves as maladaptive strategies to express, control, and transform intense emotional experiences when individuals confront intense emotional distress (34). When emotion dysregulation interacts with depressive symptoms, it may amplify negative affect, thereby increasing NSSI severity. Accordingly, adolescents engaging in NSSI typically exhibit a profile marked by early adversity, environmental stressors, emotion dysregulation, and persistent emotional distress (35).

Cumulative evidence links childhood maltreatment to emotion dysregulation (36). However, most existing studies have treated maltreatment types as a latent variable rather than examining the distinct contributions of individual types. Fares-Otero et al. (37) observed that global childhood maltreatment and each individual maltreatment form correlate with compromised emotion regulation, with emotional neglect yielding the largest effect. Christ et al. (38) further demonstrated that only emotional abuse independently predicts emotion dysregulation. In line with this, Dagnino et al. (39) identified emotional abuse as the leading trauma-related risk factor for emotion dysregulation among depressed patients.

Among individuals with major depressive disorder (MDD), CM contributes to emotion dysregulation, which in turn exacerbates depressive symptom severity (40). Individuals with depressive disorders exhibit significantly greater emotion dysregulation (41). Emotion dysregulation significantly predicts depression severity over and above social functioning and demographic covariates (42). From a developmental perspective, early childhood maltreatment has been linked to greater emotion dysregulation (43). Longitudinal evidence further indicates that emotion regulation difficulties in early adolescence predict subsequent depressive symptoms (44).

Emotion dysregulation mediated the pathway from CM to NSSI. Among adolescent psychiatric inpatients, emotion dysregulation mediated the effects of physical and emotional abuse but not sexual abuse in relation to NSSI (45). By contrast, only emotional neglect showed a significant mediating effect, whereas other maltreatment types did not (46). These discrepancies suggest that emotion dysregulation as a mediator may vary across maltreatment types, yet few studies have systematically compared these pathways across all maltreatment types in clinical depressed adolescents. Notably, emotion regulation-focused interventions can reduce self-injurious behaviors among adolescents (47).

In summary, three critical gaps persist. First, most prior investigations either aggregate all maltreatment types into a single latent construct or analyze individual forms separately, failing to adjust for co-occurring maltreatment exposures (48). Second, the sequential mediating roles of emotion dysregulation and depressive symptoms within a unified framework remain unclear. Third, the bulk of available evidence is drawn from community adolescent cohorts, and it remains unknown whether these pathways extend to clinical depressed adolescents, who carry markedly higher NSSI risk (49).

The present study addresses these gaps by testing two hypotheses: (1) childhood maltreatment types influence NSSI sequentially through emotion dysregulation and depressive symptoms; and (2) the magnitude and patterns of effects through this serial pathway differ across the five maltreatment types. Elucidating these differential mechanisms could facilitate the design of stratified, targeted clinical interventions for maltreated adolescents with depressive disorders.

## 2 Materials and methods

### 2.1 Participants

At the psychiatric outpatient clinic of Wenzhou People’s Hospital, convenience sampling was employed to recruit 426 adolescents diagnosed with depressive disorders. Inclusion criteria were (1) aged 12–18 years, and (2) diagnosed with depressive disorders from an experienced psychiatrist based on ICD-11, and (3) capable of understanding questionnaire items and providing reliable self-reported responses. Prior to survey administration, written informed consent was secured from all participating adolescents and their parents or guardians. Adolescents were excluded if unable to complete all study assessments, or if they had manic episodes, intellectual disability, substance use disorders, or severe traumatic brain injury.

Ethical approval was granted by the Ethics Committee of Wenzhou People’s Hospital (approval number: KY-202511-005). The study was performed in accordance with the Declaration of Helsinki.

### 2.2 Procedures

Following consent acquisition, research assistants delivered standardized instructions for self-report measure completion. Trained researchers collected demographic data via one-on-one interviews, whereas adolescents independently filled out all questionnaires, which were collected onsite immediately upon completion. Any survey containing substantial missing values, contradictory or invalid answers, or implausibly fast completion durations was excluded from all analyses. Among the 450 distributed questionnaires, 426 were classified as valid, yielding a valid response rate of 94.7%.

### 2.3 Measures

#### 2.3.1 Childhood Trauma Questionnaire-Short Form (CTQ-SF)

The CTQ-SF (8), a 28-item self-report scale evaluating five maltreatment types, emotional neglect, physical neglect, emotional abuse, physical abuse, and sexual abuse, was adopted to measure childhood maltreatment in this study. Each item is scored on a 5-point Likert scale (1 = never, 5 = very often). As each subscale consists of five items, possible scores for any single domain span 5 to 25, with higher scores reflecting greater maltreatment severity. For each subscale, scores of ≥ 15 (EN), ≥ 10 (PN), ≥ 13 (EA), ≥ 10 (PA), and ≥ 8 (SA) were adopted to indicate clinically significant maltreatment, in line with previously reported cutoffs. The Chinese CTQ-SF has demonstrated sound psychometric properties in adolescent populations (50). In the present sample, all subscales yielded satisfactory internal consistency, Cronbach’s α coefficients were 0.85 (EN), 0.76 (PN), 0.82 (EA), 0.80 (PA), and 0.75 (SA).

#### 2.3.2 Difficulties in Emotion Regulation Scale-16 (DERS-16)

The DERS-16 (31), a 16-item self-report instrument capturing five subscales, clarity, impulse, non-acceptance, strategies, and goals, was adopted to measure emotion dysregulation in this study. Each item is scored on a 5-point Likert scale (0 = almost never, 4 = almost always), yielding total scores between 0 and 64, with higher scores reflecting more severe emotion dysregulation. Across diverse Chinese samples, including adolescents, college students, community adults, and male prisoners, the DERS-16 has yielded Cronbach’s α values of 0.93 to 0.96 (51). In this study, the DERS-16 yielded a Cronbach’s α of 0.87, reflecting good internal consistency.

#### 2.3.3 Symptom Checklist-90 (SCL-90) Depression Subscale

The depression subscale of the SCL-90 (52) was adopted to measure depressive symptoms in this study. The SCL-90 is a 90-item self-report checklist covering 10 symptom dimensions. Each item is scored on a 5-point Likert scale (1 = not at all, 5 = extremely), with the mean of the 13 items serving as an index of depressive symptom severity. Higher mean scores denote greater depressive symptoms severity. Consistent with prior evidence supporting the reliability of the Chinese SCL-90 depression subscale (53), good internal consistency was found for this subscale in our study (Cronbach’s α = 0.90).

#### 2.3.4 Functional Assessment of Self-Mutilation (FASM)

NSSI was assessed using the method checklist of FASM (54). The Chinese version of the FASM was adapted for Chinese clinical adolescents, consisting of method checklist and function checklist (55). The C-FASM method checklist has demonstrated favorable content and structural validity in Chinese clinical adolescent samples. Internal consistency was acceptable (Cronbach’s α = 0.81), two-week test-retest reliability was adequate (r = 0.79). In this study, only the 10-item method checklist was administered, evaluating both the frequency and versatility of NSSI behaviors. Scoring adhered to the original FASM protocol. Participants recalled the frequency with which each self-injurious behavior occurred over the preceding 12 months, with frequency scores recorded for each item. Two NSSI indicators were derived from the checklist: (1) NSSI versatility, the number of distinct self-injury methods endorsed (range: 0–10); and (2) total NSSI frequency, the sum of frequency scores across all 10 items.

### 2.4 Statistical analysis

Data were analyzed using SPSS 27.0 and Mplus 8.3. Prior to main analyses, Shapiro–Wilk normality tests were performed on all continuous study variables (see **Supplementary Table S1**). Emotion dysregulation and depressive symptoms followed normal distributions (all P > 0.05), whereas all other variables deviated significantly from normality (all P < 0.05). The raw NSSI frequency exhibited extreme skewness (7.24) and kurtosis (60.27), exceeding conventional thresholds for severe non-normality (|skewness| > 3, |kurtosis| > 10) (56). The raw NSSI frequency was therefore recoded into an 11-point ordinal scale (0, 1, 2, …, ≥10), following established practice (57, 58). This frequency-band approach addresses overdispersion and the overabundance of zeros in NSSI count data. The 11-category ordinal indicator was treated as approximately continuous rather than categorical (59). The full distribution and proportions across recoded categories are reported in **Supplementary Table S2**.

NSSI severity was modeled as a reflective latent construct with two indicators: NSSI versatility (range 0–10) and recoded NSSI frequency (0, 1, 2, …, ≥10). This specification is based on evidence that NSSI severity is multidimensional and that combining frequency and versatility yields a more accurate representation than either indicator alone (60, 61). The factor loading of NSSI versatility was fixed to 1 for identification, with factor variance freely estimated. Standardized factor loadings, residual variances, factor variance, and composite reliability are reported in the Results section.

Given the non-normal distributions, bivariate associations were examined using Kendall’s tau-b rank correlations. Multicollinearity was assessed using VIF and tolerance statistics.

Structural equation modeling was performed using Mplus 8.3. The model included the five maltreatment types as observed predictors, emotion dysregulation and depressive symptoms as serial mediators, and NSSI severity as the latent outcome. Age, sex, household income, and residence were included as *a priori* covariates (62, 63). Given the non-normal distributions of most study variables and the approximately continuous nature of the recoded NSSI frequency (11 categories), the model was estimated using the MLR estimator. Model fit was evaluated using the Satorra–Bentler scaled χ², df, CFI, TLI, RMSEA (90% CI), and SRMR. R^2^ values were reported for all endogenous variables (emotion dysregulation, depressive symptoms, and NSSI severity).

Mediation effects were tested using MLR-based robust 95% confidence intervals (delta method), with significance defined as a 95% CI excluding zero. To examine the robustness of the mediator order, two alternative models were tested: (a) a reversed-order model and (b) a correlated-mediator model. Model comparisons used AIC and BIC (64).

To evaluate robustness, sensitivity analyses were conducted using (a) raw untransformed NSSI frequency (MLR), (b) a binary NSSI outcome (WLSMV, 5,000 bootstrap resamples), and (c) composite abuse and neglect predictors. Benjamini-Hochberg FDR correction was prespecified for the 15 specific indirect effects, with q < 0.05 considered significant.

## 3 Results

### 3.1 Participant demographic and clinical characteristics

Of the 426 adolescents in the final analysis, 72.1% reported at least one type of CM. EN was the most commonly reported type (45.5%), followed by PN (41.1%), EA (38.7%), PA (16.7%), and SA (10.1%). Moreover, 66.2% reported at least one NSSI behavior over the preceding 12 months. The sample’s demographic and clinical characteristics are given in **Table 1**.

**Table 1:** Demographic and clinical characteristics of adolescents, stratified by NSSI status.

Variable	NSSI status		t/χ²/Z	P
	With NSSI (N=282)	Without NSSI (N=144)		
Demographics				
Age(years)	14.79 ± 1.90	14.63 ± 1.99	t=0.838	0.402
Sex, n (%)			χ²=9.841	0.002
Male	129 (45.7%)	89 (61.8%)		
Female	153 (54.3%)	55 (38.2%)		
Residence, n (%)			χ²=1.308	0.253
Rural	116 (41.1%)	51 (35.4%)		
Urban	166 (58.9%)	93 (64.6%)		
Childhood maltreatment				
EN	15.00 (12.00, 18.00)	12.00 (9.00, 16.00)	Z = 6.476	< 0.001
PN	10.00 (7.00, 12.00)	7.00 (6.00, 10.00)	Z = 6.640	< 0.001
EA	12.00 (9.00, 15.00)	9.00 (7.00, 12.00)	Z = 6.252	< 0.001
PA	7.00 (5.00, 9.00)	5.00 (5.00, 7.00)	Z=6.391	< 0.001
SA	5.00 (5.00, 6.00)	5.00 (5.00, 5.00)	Z=4.797	< 0.001
Psychosocial variables				
emotion dysregulation	36.07 ± 10.35	26.42 ± 9.52	t=9.349	< 0.001
Depression	3.59 ± 0.61	3.06 ± 0.58	t=8.741	< 0.001
NSSI characteristics				
NSSI versatility	3.00 (2.00, 4.00)	0.00 (0.00, 0.00)	Z=17.310	< 0.001
NSSI frequency	6.00 (5.00, 8.00)	0.00 (0.00, 0.00)	Z=17.310	< 0.001

NSSI, non-suicidal self-injury; EN, emotional neglect; PN, physical neglect; EA, emotional abuse; PA, physical abuse; SA, sexual abuse; Data are presented as mean ± SD (t-test), n (%) (χ² test), or median (P25, P75) (Mann-Whitney U test).

### 3.2 Bivariate correlations and preliminary analyses

Kendall’s tau-b correlations were computed, with detailed results shown in **Table 2**. All study variables were positively and significantly correlated (all P <.01). All VIF values were below 5 (range: 1.008–2.449), and tolerance values exceeded 0.40 (range: 0.408–0.993), indicating no severe multicollinearity.

**Table 2:** Kendall’s tau-b correlations among study variables.

Variables	1	2	3	4	5	6	7	8	9
1. EN	1								
2. PN	.245**	1							
3. EA	.185**	.216**	1						
4. PA	.288**	.243**	.244**	1					
5. SA	.243**	.245**	.342**	.357**	1				
6. DERS	.335**	.354**	.323**	.208**	.220**	1			
7. Depression	.347**	.264**	.318**	.245**	.247**	.531**	1		
8. NSSI versatility	.268**	.240**	.283**	.281**	.186**	.380**	.383**	1	
9. NSSI frequency	.280**	.258**	.296**	.298**	.228**	.389**	.355**	.707**	1

EN, emotional neglect; PN, physical neglect;EA, emotional abuse; PA, physical abuse; SA, sexual abuse; ED, emotion dysregulation; NSSI, non-suicidal self-injury.

** indicates P < 0.01.

Chi-square tests and independent-samples t-tests were employed for preliminary demographic comparisons to examine demographic differences across key study variables. Chi-square results indicated significant sex-based differences in binary NSSI grouping (χ² = 9.841, P = 0.002), with females comprising a greater share of participants with NSSI histories. Urban and rural adolescents with depressive disorders differed significantly on emotion dysregulation (t = −2.112, P = 0.035) and depressive symptom severity (t = −2.219, P = 0.027).

### 3.3 Path model results

The latent NSSI construct demonstrated strong psychometric properties. Standardized factor loadings were 0.904 (95% CI [0.859, 0.950]) for NSSI versatility and 0.849 (95% CI [0.802, 0.896]) for NSSI frequency, with residual variances of 0.182 and 0.279, respectively. The unstandardized factor variance was 1.918 (SE = 0.209), with the NSSI versatility loading fixed to 1 for identification. Composite reliability (ω) was 0.87.

The serial mediation model, estimated with the MLR estimator, demonstrated excellent fit to the data: robust χ² (10) = 14.923, P = 0.135; CFI = 0.995; TLI = 0.981; RMSEA = 0.034 (90% CI [0.000-0.068], P-close = 0.750); and SRMR = 0.013 (**Figure 1**). R^2^ values were 0.430 for emotion dysregulation, 0.552 for depressive symptoms, and 0.483 for NSSI severity, indicating substantial explanatory power for all three endogenous variables. **Figure 2** displays the standardized path coefficients for the final model (only significant paths are shown, P < 0.05). Direct and specific indirect effects are presented in **Table 3**; total indirect and total effects for each maltreatment type are summarized in **Table 4**.

**Figure 1:**
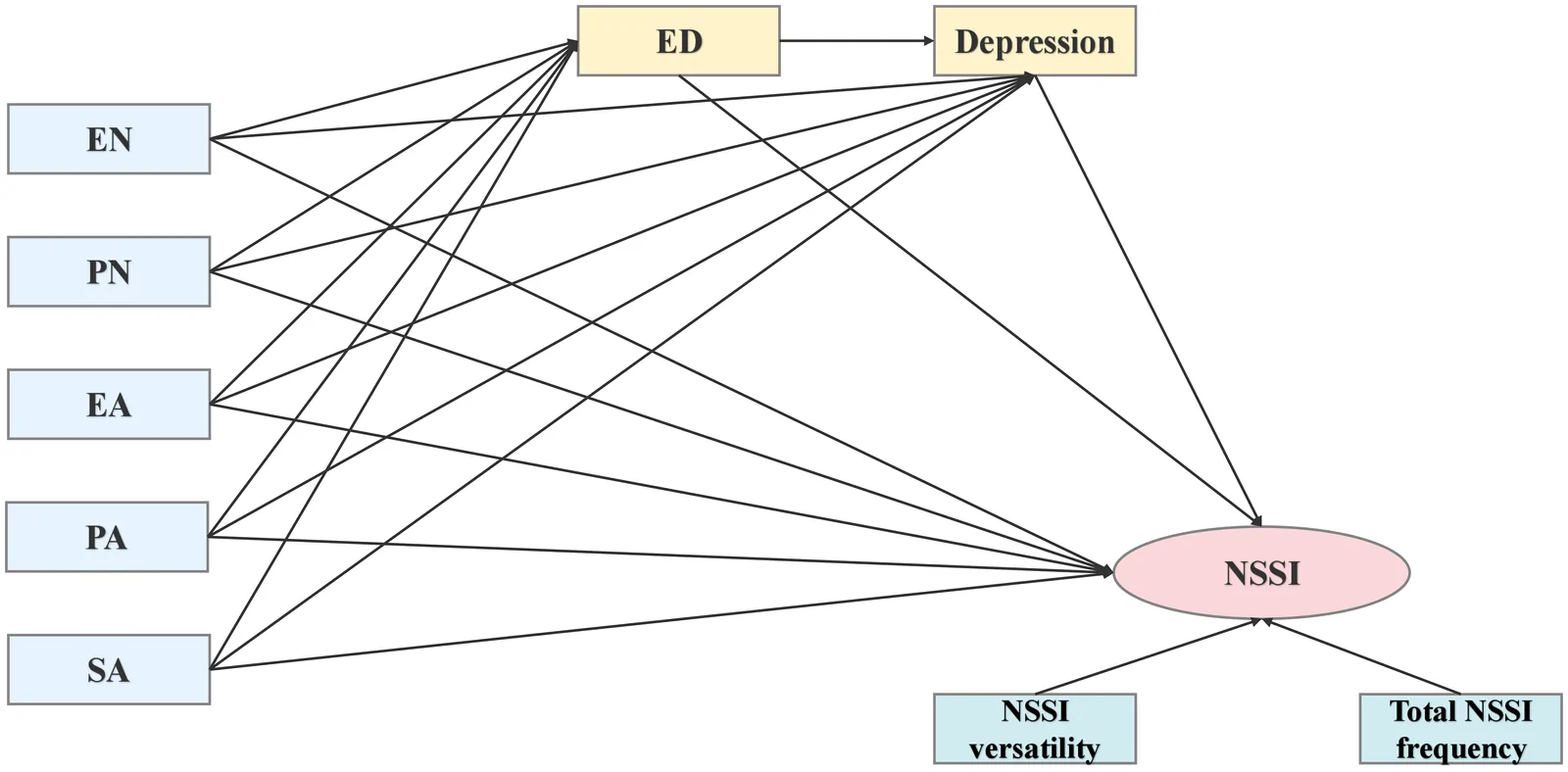
Hypothesized serial mediation model. EN, emotional neglect; PN, physical neglect;EA, emotional abuse; PA, physical abuse; SA, sexual abuse; ED, emotion dysregulation; NSSI, non-suicidal self-injury.

**Figure 2:**
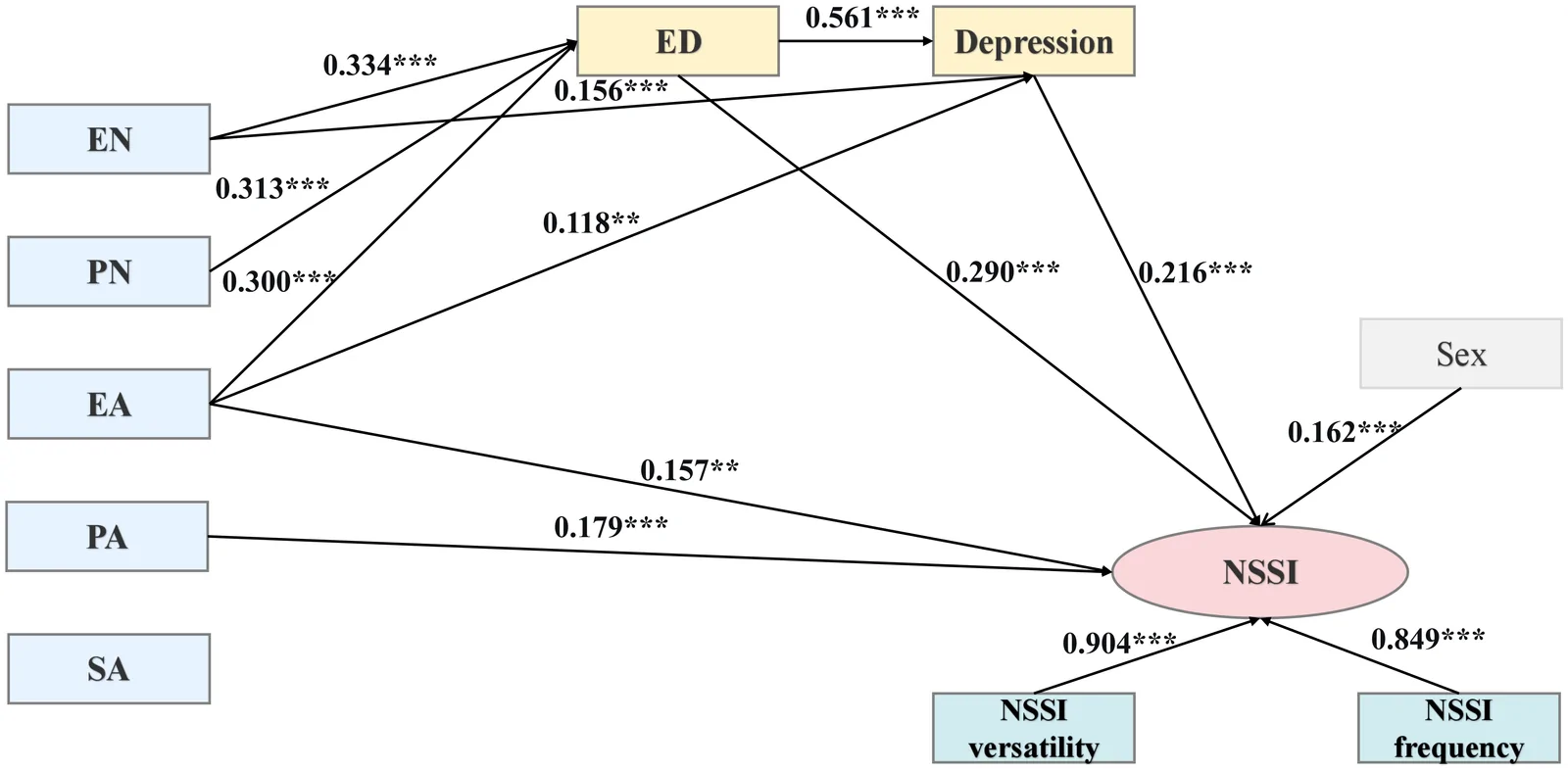
Serial mediation model with standardized path coefficients. For clarity, non-significant paths are not shown. EN, emotional neglect; PN, physical neglect; EA, emotional abuse; PA, physical abuse; SA, sexual abuse; ED, emotion dysregulation; NSSI, non-suicidal self-injury. ** indicates P < 0.001.

**Table 3:** Direct and indirect effects of maltreatment types on NSSI.

Path	β	SE	95% CI	P	PM %
Direct effects					
EA → NSSI	0.157	0.046	[0.067, 0.246]	0.001	–
PA → NSSI	0.179	0.048	[0.085, 0.274]	< 0.001	–
EN → NSSI	0.061	0.053	[-0.043, 0.166]	0.248	–
PN → NSSI	0.033	0.048	[-0.060, 0.127]	0.487	–
SA → NSSI	-0.052	0.049	[-0.148, 0.045]	0.297	–
ED → NSSI	0.290	0.061	[0.170, 0.411]	< 0.001	–
Depression → NSSI	0.216	0.056	[0.106, 0.327]	< 0.001	–
ED → Depression	0.561	0.042	[0.479, 0.642]	< 0.001	–
Indirect effects					
EA → ED → NSSI	0.087	0.022	[0.043, 0.131]	< 0.001	28
EA → Depression → NSSI	0.026	0.011	[0.004, 0.047]	0.021	8
EA → ED → Depression → NSSI	0.036	0.011	[0.015, 0.058]	0.001	12
EN → ED → NSSI	0.097	0.024	[0.049, 0.145]	< 0.001	42
EN → Depression → NSSI	0.034	0.013	[0.008, 0.059]	0.009	15
EN → ED → Depression → NSSI	0.041	0.011	[0.018, 0.063]	< 0.001	18
PN → ED → NSSI	0.091	0.022	[0.047, 0.135]	< 0.001	58
PN → Depression → NSSI	-0.006	0.009	[-0.023, 0.011]	0.458	–
PN → ED → Depression → NSSI	0.038	0.012	[0.015, 0.061]	0.001	24
PA → ED → NSSI	-0.016	0.013	[-0.042, 0.010]	0.223	–
PA → Depression → NSSI	0.016	0.009	[-0.002, 0.034]	0.088	–
PA → ED → Depression → NSSI	-0.007	0.006	[-0.018, 0.004]	0.236	–
SA → ED → NSSI	-0.012	0.011	[-0.034, 0.010]	0.295	–
SA → Depression → NSSI	0.010	0.009	[-0.008, 0.027]	0.271	–
SA → ED → Depression → NSSI	-0.005	0.005	[-0.014, 0.004]	0.293	–

Standardized coefficients from the MLR model. 95% CI = MLR-based robust confidence interval (delta method). PM%, proportion mediated, calculated as the standardized specific indirect effect divided by the standardized total effect, and reported only for statistically significant specific indirect effects. ED, emotion dysregulation; NSSI, non-suicidal self-injury; EA, emotional abuse; EN, emotional neglect; PN, physical neglect; PA, physical abuse; SA, sexual abuse.

**Table 4:** Total indirect and total effects for all maltreatment types.

Maltreatment type	Total indirect effect	95% CI	P	Total effect	95% CI	P
EN → NSSI	0.171	[0.119, 0.223]	< 0.001	0.233	[0.133, 0.332]	< 0.001
PN → NSSI	0.122	[0.074, 0.171]	< 0.001	0.156	[0.062, 0.249]	0.001
EA → NSSI	0.149	[0.094, 0.204]	< 0.001	0.306	[0.218, 0.393]	< 0.001
PA → NSSI	-0.007	[-0.048, 0.034]	0.752	0.173	[0.066, 0.279]	0.002
SA → NSSI	-0.007	[-0.042, 0.027]	0.686	-0.059	[-0.168, 0.051]	0.292

Standardized coefficients from the MLR model. NSSI, non-suicidal self-injury; EN, emotional neglect; PN, physical neglect; EA, emotional abuse; PA, physical abuse; SA, sexual abuse.

Emotional abuse exerted both a significant direct effect (β = 0.157, 95% CI [0.067, 0.246], P = 0.001) and a significant total indirect effect (β = 0.149, 95% CI [0.094, 0.204], P < 0.001) on NSSI severity. Three specific indirect pathways were significant: EA → ED → NSSI (β = 0.087, 95% CI [0.043, 0.131], P < 0.001), EA → Depression → NSSI (β = 0.026, 95% CI [0.004, 0.047], P = 0.021), and EA → ED → Depression → NSSI (β = 0.036, 95% CI [0.015, 0.058], P = 0.001).

Emotional neglect showed no significant direct effect (β = 0.061, 95% CI [−0.043, 0.166], P = 0.248) but a significant total indirect effect (β = 0.171, 95% CI [0.119, 0.223], P < 0.001). All three specific indirect pathways were significant: EN → ED → NSSI (β = 0.097, 95% CI [0.049, 0.145], P < 0.001), EN → Depression → NSSI (β = 0.034, 95% CI [0.008, 0.059], P = 0.009), and EN → ED → Depression → NSSI (β = 0.041, 95% CI [0.018, 0.063], P < 0.001).

Physical neglect similarly showed no significant direct effect (β = 0.033, 95% CI [−0.060, 0.127], P = 0.487) but a significant total indirect effect (β = 0.122, 95% CI [0.074, 0.171], P < 0.001). The indirect pathways through emotion dysregulation (β = 0.091, 95% CI [0.047, 0.135], P < 0.001) and through the serial ED → Depression chain (β = 0.038, 95% CI [0.015, 0.061], P = 0.001) were significant, whereas the pathway through depressive symptoms alone was not (β = −0.006, 95% CI [−0.023, 0.011], P = 0.458).

Physical abuse showed a significant direct effect (β = 0.179, 95% CI [0.085, 0.274], P < 0.001) but no significant total indirect effect (β = −0.007, 95% CI [−0.048, 0.034], P = 0.752). Sexual abuse showed neither a significant direct effect (β = −0.052, 95% CI [−0.148, 0.045], P = 0.297) nor a significant total indirect effect (β = −0.007, 95% CI [−0.042, 0.027], P = 0.686).

Across all significant specific indirect pathways, the proportion mediated through emotion dysregulation ranged from 28% for EA to 58% for PN, indicating that emotion dysregulation was a substantial mechanism linking childhood maltreatment to NSSI.

Within the overall model, the path from emotion dysregulation to depressive symptoms was the strongest (β = 0.561, 95% CI [0.479, 0.642], P < 0.001), followed by the path from emotion dysregulation to NSSI (β = 0.290, 95% CI [0.170, 0.411], P < 0.001) and the path from depressive symptoms to NSSI (β = 0.216, 95% CI [0.106, 0.327], P < 0.001). Regarding covariates, sex significantly predicted NSSI severity (β = 0.162, 95% CI [0.085, 0.240], P < 0.001), with female adolescents reporting higher NSSI severity. Age (β = 0.041, P = 0.301), residence (β = −0.069, P = 0.100), and household income (β = 0.050, P = 0.175) showed non-significant effects.

Formal pairwise contrasts of the total indirect effects showed no significant differences among EA, EN, and PN (all P > 0.05), whereas PA and SA had significantly weaker total indirect effects than EA, EN, and PN (all P < 0.05; **Supplementary Table S7**). PA and SA did not differ from each other (P = 0.864).

Sensitivity analyses using raw NSSI frequency (**Supplementary Table S3**), binary NSSI (**Supplementary Table S4**), and composite abuse/neglect predictors (**Supplementary Table S5**) were largely consistent with the primary model, with emotion dysregulation remaining a significant mediator for emotional abuse and neglect. After Benjamini-Hochberg FDR correction, 8 of the 15 prespecified specific indirect effects remained significant at q < 0.05 (**Supplementary Table S6**). Alternative model tests showed that the reversed-order and correlated-mediator models yielded identical fit to the hypothesized model (65), whereas a parallel mediation model without a path between the two mediators showed substantially poorer fit (**Supplementary Table S8**).

## 4 Discussion

This study examined how five CM types differentially affect NSSI via emotion dysregulation and depressive symptoms in adolescents with depressive disorders. Focusing on this clinical population confers distinct empirical significance, as adolescents with depression demonstrate markedly higher NSSI prevalence (3) and more pronounced emotion regulatory deficits relative to community-based youth (66), highlighting the necessity of delineating maltreatment-specific pathogenic pathways within this high-risk group. The present findings revealed heterogeneous pathway patterns across maltreatment types. Emotional abuse predicted NSSI through both direct and indirect routes, supporting a dual-pathway mechanism. In contrast, emotional and physical neglect indirectly affected NSSI via emotion dysregulation and depressive symptoms, indicating full mediation. Physical abuse was associated with NSSI solely through a direct pathway, while sexual abuse showed no significant predictive effect after simultaneous adjustment for all other maltreatment types and mediating variables. Emotion dysregulation served as a shared intermediate mechanism for emotional abuse and neglect, but not for physical abuse or sexual abuse. Formal contrasts further showed that the total indirect effects of EA, EN, and PN did not differ significantly, whereas PA and SA showed significantly weaker total indirect effects. This shared mediating role for emotional abuse and neglect aligns with meta-analytic evidence documenting emotion regulatory deficits as a core vulnerability factor for diverse psychopathologies (67). Notably, such mechanistic pathways may be particularly salient among clinical depressed adolescents, whose regulatory functioning is already compromised by severe depressive symptoms, further underscoring the clinical centrality of emotion dysregulation in NSSI.

In our clinical sample of adolescents with depressive disorders, path analysis further corroborates prior empirical evidence identifying emotional abuse as a common contributor to emotion dysregulation (39), depressive symptoms, and NSSI (68). For emotional abuse, the ED-mediated pathway accounted for 28% of the total effect (**Table 3**). Notably, the direct path from emotional abuse to NSSI remained significant after accounting for the mediators (β = 0.157), indicating partial mediation and confirming that emotion dysregulation and depression collectively but incompletely account for this predictive association. Emotion dysregulation emerged as a critical intermediary linking emotional abuse and neglect to adolescent NSSI, but not for physical abuse or sexual abuse. Specifically, exposure to emotional abuse elevates emotion regulatory deficits, which sequentially exacerbate depressive symptoms and further increase NSSI susceptibility. Consistent with previous research focusing on mood-disordered adolescents (69), emotional abuse exerts dual direct and indirect effects on NSSI via disrupted emotional processing. Importantly, the present findings further validate and extend the classic emotion regulation model of NSSI (34) by consolidating its applicability specifically within clinical adolescent populations with depressive disorders.

Emotional abuse, a severe form of early relational trauma, involves chronic hostile and devaluing caregiver-child interactions (e.g., verbal humiliation and belittlement) that undermine adolescents’ self-worth and psychological integrity (70). Through repeated exposure to such maltreatment, children gradually internalize negative cognitive schemas, such as feeling stupid, worthless, or unlovable (71), and develop a disrupted sense of self-identity (72). These cognitive distortions, in turn, impair the maturation of core emotion regulation capacities, compromising the individual’s ability to recognize and modulate affective responses in subsequent social contexts, thereby fostering socioemotional maladjustment and accelerating the development of depressive symptoms. When emotion dysregulation co-occurs with relevant depressive symptoms, NSSI may be adopted as a maladaptive but immediately effective strategy for alleviating overwhelming negative affect (73). Emotional abuse not only increases the likelihood of NSSI onset but also impedes adolescents’ capacity to disengage from self-injurious behaviors over time (74), This is particularly relevant for clinical depressed adolescents, whose emotion regulation is often compromised.

Convergent neuroimaging evidence suggests that childhood emotional abuse selectively impairs the prefrontal-limbic social regulatory network, particularly attenuating prefrontal top-down inhibitory control over amygdala-mediated processing of negative social cues (75, 76). This framework provides a direct neurocircuitry foundation for the pervasive emotion dysregulation and subsequent depressive symptoms observed in affected adolescents. When this core regulatory pathway becomes functionally exhausted, NSSI may emerge as a somatic compensatory mechanism. Specifically, self-inflicted pain activates the endogenous opioid system, which transiently suppresses amygdala hyperreactivity (77) and serves as a critical biological brake against overwhelming negative affect. Future research using fMRI and EEG could further clarify the neural mechanisms underlying the sequential pathway.

Emotional neglect (45.5%) and physical neglect (41.1%) were the top two maltreatment types in our clinical sample, yet neither directly predicted NSSI. Both types exerted their influence indirectly, through the serial mediation. Significant total indirect effects emerged for both EN (β = 0.171) and PN (β = 0.122). This indirect-only pattern is consistent with prior findings. Emotional neglect was found to influence NSSI solely through indirect pathways (20), and a similar pattern was observed for physical neglect (21). In a large Chinese community sample, neglect predicted NSSI primarily through depressive symptoms (86.5%) (29). Emotional neglect exhibited a similar indirect-only effect among Chinese adolescents with MDD (78). Although the specific mediators varied across these studies (e.g., mindfulness, depression, impulsivity), the common finding is that neglect does not directly predict NSSI; its effects are consistently indirect.

This pattern likely reflects the defining feature of neglect as an act of omission (79): caregivers chronically fail to provide the basic care and developmental stimulation required for children’s physical and emotional development. This distinguishes neglect fundamentally from abuse, which entails active hostile acts (80). Because neglect deprives rather than attacks, it selectively impairs antecedent-focused emotion regulation, the capacity to identify, interpret, and modulate internal affective states (81). These emotion-processing deficits sequentially heighten depressive symptoms, which in turn increase NSSI risk (82). Neuroimaging evidence converges on this interpretation: neglect disrupts prefrontal–limbic circuitry underlying emotion recognition and interoceptive processing, whereas abuse preferentially activates acute threat-related stress responses (83). Neglect thus lacks a direct motivational pathway to NSSI in adolescents with depressive disorders. Its harm unfolds indirectly through the progressive erosion of core socioemotional functions, with emotion dysregulation as the primary mechanism.

Physical abuse exhibited a distinct predictive pattern relative to emotional abuse and neglect, showing a direct effect on NSSI (β = 0.179) with no significant indirect effects, a finding consistent with Yang et al. (19). This study extends this observation to clinical depressed adolescents. In Chinese cultural contexts, corporal punishment has historically been normalized as a disciplinary practice (84). This normalization may obscure the pathological harm of physical abuse, as children’s socioemotional needs are frequently overshadowed by behavioral compliance (85). Physical abuse may bypass emotional-cognitive integration and directly activate somatic stress responses. Diez et al. (86) found that physical abuse exposure enhances amygdala-motor and insula-motor connectivity, suggesting a possible aberrant coupling between limbic and motor regions. Such a link may, in turn, directly translate emotional distress into NSSI.

Although bivariate correlations revealed significant positive associations between sexual abuse and both NSSI indicators, it exerted no significant direct or indirect effects in the full SEM after controlling for all five maltreatment types and the two mediators (all P > 0.05). This pattern aligns with prior evidence, including meta-analytic findings that sexual abuse accounts for negligible unique variance in self-injury after adjusting for co-occurring trauma and internalizing psychopathology (18), as well as in clinical samples, where only emotional abuse (not sexual abuse) remained directly associated with NSSI after adjusting for all maltreatment types (17). Drawing on poly-victimization theory (48), this null effect may be explained by the clinical characteristics of our sample. Psychiatric outpatients with depressive disorders rarely experience sexual abuse in isolation; rather, sexual abuse is rarely reported when emotional abuse and neglect are not also present among psychiatric outpatients with depressive disorders (87). Accordingly, once emotional abuse and neglect are adjusted for, the bivariate association of SA with NSSI diminishes, with little independent predictive effect remaining. One methodological caveat is that our study did not consider trauma-specific sequelae of sexual victimization, such as dissociation or PTSD symptoms, which may obscure potential indirect pathways.

Notably, sex significantly predicted NSSI, with higher self-injury scores in females. This sex difference aligns with previous meta-analyses reporting greater NSSI vulnerability among females (88).

The model explained 43.0% of the variance in emotion dysregulation, 55.2% of the variance in depressive symptoms, and 48.3% of the variance in NSSI severity. These values indicate that the proposed pathways have substantial explanatory value beyond statistical significance alone. However, these cross-sectional estimates cannot establish causality.

This study has several limitations. First, emotion regulation capacity and NSSI behaviors vary with age (89, 90), and our sample was limited to adolescents; whether these findings extend to other age groups remains to be tested. Second, because we used convenience sampling at a single outpatient psychiatric clinic in China, the generalizability of our findings to other socioeconomic or cultural groups remains uncertain. Third, causal inferences regarding the temporal sequence of the mediation pathways cannot be drawn from a cross-sectional design; alternative models fit equally well, leaving the ED–depression ordering undetermined. Longitudinal studies are needed to establish temporal precedence. Fourth, single-timepoint self-report measures may introduce common method variance, which cannot be fully excluded despite validated instruments and latent variable modeling of NSSI. Future research should use multi-method, multi-informant designs. Fifth, a recent meta-analysis showed that retrospective self-reports of childhood maltreatment yielded stronger associations with psychopathology (OR = 2.21) than did objective records (OR = 1.56) (91), implying that self-report measures may overestimate the true effect sizes. Future studies using prospective designs or neurophysiological measures could help distinguish between subjective recall and objective exposure. Finally, NSSI severity was modeled with only two indicators, which is less robust than models with three or more, though factor loadings and reliability were acceptable; future research should add more indicators (e.g., recency).

## 5 Conclusion

Collectively, these results demonstrate that different CM types predict NSSI via heterogeneous pathways among adolescents with depressive disorders. Emotional abuse showed both direct and indirect effects, whereas neglect showed indirect-only effects. Physical abuse displayed an exclusive direct effect, and sexual abuse showed no independent predictive effect. Emotion dysregulation served as a shared mediating mechanism for emotional abuse and neglect, but not for physical abuse or sexual abuse. Formal contrasts indicated that the total indirect effects of EA, EN, and PN did not differ significantly, whereas PA and SA showed significantly weaker total indirect effects. These findings highlight the value of type-informed trauma risk assessment and suggest that interventions targeting emotion dysregulation may be promising for maltreated adolescents with depressive disorders. Further longitudinal studies should confirm the temporal ordering of these pathways.

## Data Availability

The raw data supporting the conclusions of this article will be made available by the authors, without undue reservation.
