# Supplementary material for: Differential pathways from childhood maltreatment types to non-suicidal self-injury in adolescents with depressive disorders: a serial mediation model
Source: Front Psychiatry. 2026 Sep 30;17:1960956. doi: 10.3389/fpsyt.2026.1960956 (PMC13661984; doi:10.3389/fpsyt.2026.1960956)
Supplement: Supplementary file 1 [file Table1.docx]

Supplementary Material

# 1 Supplementary Tables

**Supplementary Table S1.** Descriptive Statistics and Normality Tests for All Study Variables

| **Variable** | **Mean** | **SD** | **Skewness** | **Kurtosis** | **Shapiro–Wilk W** | **P** |
| --- | --- | --- | --- | --- | --- | --- |
| EN | 14.20 | 4.77 | 0.02 | -0.56 | 0.984 | < 0.001 |
| PN | 8.91 | 3.11 | 0.43 | -0.66 | 0.934 | < 0.001 |
| EA | 11.24 | 4.05 | 0.33 | -0.30 | 0.968 | < 0.001 |
| PA | 7.15 | 2.62 | 1.55 | 3.19 | 0.800 | < 0.001 |
| SA | 5.78 | 1.38 | 2.26 | 5.20 | 0.629 | < 0.001 |
| ED | 32.81 | 11.05 | 0.16 | -0.07 | 0.995 | 0.185 |
| Depression | 3.41 | 0.65 | -0.07 | 0.01 | 0.995 | 0.188 |
| NSSI versatility | 2.11 | 2.13 | 0.85 | 0.01 | 0.872 | < 0.001 |
| NSSI frequency (transformed) | 4.73 | 3.74 | -0.16 | -1.44 | 0.844 | < 0.001 |
| Raw NSSI frequency | 28.98 | 123.35 | 7.24 | 60.27 | 0.223 | < 0.001 |

Note. EN = emotional neglect; PN = physical neglect; EA = emotional abuse; PA = physical abuse; SA = sexual abuse; ED = emotion dysregulation.

**Supplementary Table S2.** Distribution of raw and recoded NSSI frequencyPanel

**A.** Raw NSSI frequency (grouped)

| **Raw NSSI frequency** | **n** | **%** | **Cumulative %** |
| --- | --- | --- | --- |
| 0 | 144 | 33.8 | 33.8 |
| 1 | 1 | 0.2 | 34.0 |
| 2 | 1 | 0.2 | 34.3 |
| 3 | 2 | 0.5 | 34.7 |
| 4 | 14 | 3.3 | 38.0 |
| 5 | 36 | 8.5 | 46.5 |
| 6 | 68 | 16.0 | 62.4 |
| 7 | 54 | 12.7 | 75.1 |
| 8 | 29 | 6.8 | 81.9 |
| 9 | 16 | 3.8 | 85.7 |
| 10–19 | 10 | 2.3 | 88.0 |
| 20–49 | 15 | 3.5 | 91.5 |
| 50–99 | 18 | 4.2 | 95.8 |
| 100–199 | 5 | 1.2 | 96.9 |
| 200–499 | 6 | 1.4 | 98.4 |
| ≥500 | 7 | 1.6 | 100.0 |
| Total | 426 | 100.0 |  |

**Note.** Raw NSSI frequency: mean = 28.98, SD = 123.35, median = 6, skewness = 7.24, kurtosis = 60.27, range = 0–1350.

**Panel B.** Recoded NSSI frequency categories (used in the primary analysis)

| **Recoded category** | **n** | **%** | **Cumulative %** |
| --- | --- | --- | --- |
| 0 | 144 | 33.8 | 33.8 |
| 1 | 1 | 0.2 | 34.0 |
| 2 | 1 | 0.2 | 34.3 |
| 3 | 2 | 0.5 | 34.7 |
| 4 | 14 | 3.3 | 38.0 |
| 5 | 36 | 8.5 | 46.5 |
| 6 | 68 | 16.0 | 62.4 |
| 7 | 54 | 12.7 | 75.1 |
| 8 | 29 | 6.8 | 81.9 |
| 9 | 16 | 3.8 | 85.7 |
| ≥10 | 61 | 14.3 | 100.0 |
| Total | 426 | 100.0 |  |

**Note**. Recoded NSSI frequency (0, 1, 2, …, ≥10): mean = 4.73, SD = 3.74, median = 6.00, skewness = −0.16, kurtosis = −1.44

**Supplementary Table S3.** Sensitivity analysis using raw (untransformed) NSSI frequency as the frequency indicator (MLR estimation)

| **Pathway** | **β** | **SE** | **95% CI** | **P** |
| --- | --- | --- | --- | --- |
| **EN to NSSI** | | | | |
| Total effect | 0.205 | 0.069 | [0.070, 0.341] | 0.003 |
| Total indirect | 0.165 | 0.034 | [0.098, 0.232] | < 0 .001 |
| EN → ED → NSSI | 0.086 | 0.031 | [0.025, 0.148] | 0.006 |
| EN → Depression → NSSI | 0.036 | 0.013 | [0.011, 0.060] | 0.005 |
| EN → ED → Depression → NSSI | 0.043 | 0.011 | [0.022, 0.064] | < 0.001 |
| Direct effect (EN → NSSI) | 0.040 | 0.056 | [−0.070, 0.151] | 0.475 |
| **PN to NSSI** | | | | |
| Total effect | 0.119 | 0.043 | [0.034, 0.203] | 0.006 |
| Total indirect | 0.114 | 0.030 | [0.056, 0.173] | < 0.001 |
| PN → ED → NSSI | 0.081 | 0.028 | [0.026, 0.136] | 0.004 |
| PN → Depression → NSSI | −0.007 | 0.009 | [−0.025, 0.011] | 0.455 |
| PN → ED → Depression → NSSI | 0.040 | 0.011 | [0.019, 0.062] | < 0.001 |
| Direct effect (PN → NSSI) | 0.004 | 0.049 | [−0.092, 0.100] | 0.931 |
| **EA to NSSI** | | | | |
| Total effect | 0.283 | 0.059 | [0.167, 0.400] | < 0.001 |
| Total indirect | 0.143 | 0.032 | [0.080, 0.207] | < 0.001 |
| EA → ED → NSSI | 0.077 | 0.028 | [0.023, 0.132] | 0.005 |
| EA → Depression → NSSI | 0.027 | 0.011 | [0.006, 0.049] | 0.014 |
| EA → ED → Depression → NSSI | 0.039 | 0.010 | [0.018, 0.059] | < 0.001 |
| Direct effect (EA → NSSI) | 0.140 | 0.048 | [0.045, 0.235] | 0.004 |
| **PA to NSSI** | | | | |
| Total effect | 0.161 | 0.053 | [0.058, 0.264] | 0.002 |
| Total indirect | −0.004 | 0.021 | [−0.045, 0.036] | 0.836 |
| PA → ED → NSSI | −0.014 | 0.012 | [−0.039, 0.010] | 0.254 |
| PA → Depression → NSSI | 0.017 | 0.010 | [−0.002, 0.036] | 0.087 |
| PA → ED → Depression → NSSI | −0.007 | 0.006 | [−0.019, 0.005] | 0.232 |
| Direct effect (PA → NSSI) | 0.165 | 0.047 | [0.073, 0.258] | < 0.001 |
| **SA to NSSI** | | | | |
| Total effect | −0.050 | 0.057 | [−0.162, 0.061] | 0.379 |
| Total indirect | −0.006 | 0.017 | [−0.039, 0.028] | 0.744 |
| SA → ED → NSSI | −0.011 | 0.010 | [−0.030, 0.009] | 0.294 |
| SA → Depression → NSSI | 0.010 | 0.009 | [−0.008, 0.029] | 0.270 |
| SA → ED → Depression → NSSI | −0.005 | 0.005 | [−0.015, 0.004] | 0.286 |
| Direct effect (SA → NSSI) | −0.045 | 0.051 | [−0.144, 0.055] | 0.382 |

**Note.** all other specifications were identical to the primary model; 95% CIs are MLR-based robust. Model fit: χ²(10) = 37.527, P < 0.001; CFI = 0.965; TLI = 0.854; RMSEA = 0.080; SRMR = 0.031. All indirect pathways remained consistent with the primary model.

**Supplementary Table S4.** Sensitivity analysis using binary NSSI (presence/absence) as outcome

| **Pathway** | **β** | **SE** | **95% CI** | **P** |
| --- | --- | --- | --- | --- |
| EN to NSSI | | | | |
| Total indirect | 0.148 | 0.032 | [0.088, 0.212] | < 0.001 |
| EN → ED → NSSI | 0.089 | 0.029 | [0.035, 0.148] | 0.002 |
| EN → Depression → NSSI | 0.027 | 0.014 | [0.003, 0.058] | 0.060 |
| EN → ED → Depression → NSSI | 0.032 | 0.015 | [0.009, 0.062] | 0.027 |
| Direct effect (EN → NSSI) | 0.050 | 0.067 | [−0.082, 0.181] | 0.460 |
| **PN to NSSI** | | | | |
| Total indirect | 0.109 | 0.027 | [0.058, 0.162] | < .001 |
| PN → ED → NSSI | 0.084 | 0.026 | [0.033, 0.135] | 0.001 |
| PN → Depression → NSSI | −0.005 | 0.008 | [−0.022, 0.006] | 0.509 |
| PN → ED → Depression → NSSI | 0.030 | 0.014 | [0.005, 0.059] | 0.031 |
| Direct effect (PN → NSSI) | 0.132 | 0.062 | [0.011, 0.253] | 0.033 |
| **EA to NSSI** | | | | |
| Total indirect | 0.129 | 0.032 | [0.071, 0.194] | < .001 |
| EA → ED → NSSI | 0.080 | 0.027 | [0.030, 0.136] | 0.003 |
| EA → Depression → NSSI | 0.020 | 0.012 | [0.002, 0.046] | 0.078 |
| EA → ED → Depression → NSSI | 0.029 | 0.013 | [0.005, 0.058] | 0.032 |
| Direct effect (EA → NSSI) | 0.110 | 0.064 | [−0.021, 0.230] | 0.087 |
| **PA to NSSI** | | | | |
| Total indirect | −0.007 | 0.019 | [−0.046, 0.030] | 0.705 |
| PA → ED → NSSI | −0.015 | 0.013 | [−0.043, 0.010] | 0.267 |
| PA → Depression → NSSI | 0.013 | 0.009 | [−0.001, 0.032] | 0.149 |
| PA → ED → Depression → NSSI | −0.005 | 0.005 | [−0.017, 0.004] | 0.317 |
| Direct effect (PA → NSSI) | 0.165 | 0.078 | [0.028, 0.330] | 0.033 |
| **SA to NSSI** | | | | |
| Total indirect | −0.007 | 0.016 | [−0.040, 0.024] | 0.657 |
| SA → ED → NSSI | −0.011 | 0.011 | [−0.036, 0.009] | 0.328 |
| SA → Depression → NSSI | 0.008 | 0.008 | [−0.006, 0.026] | 0.327 |
| SA → ED → Depression → NSSI | −0.004 | 0.004 | [−0.014, 0.004] | 0.361 |
| Direct effect (SA → NSSI) | −0.034 | 0.083 | [−0.174, 0.152] | 0.680 |

**Note.** This sensitivity analysis used binary NSSI (0 = no NSSI, 1 = any NSSI) as the outcome, estimated with the WLSMV estimator and 5,000 percentile bootstrap resamples. Standardized coefficients (STDYX) are reported; 95% CIs are percentile bootstrap confidence intervals. Because the outcome is binary, the model is saturated (df = 0) and global fit indices are not informative. The direction and significance of the core indirect pathways were consistent with the primary model.

**Supplementary Table S5.** Sensitivity analysis using combined abuse/neglect composites (MLR estimation)

| **Pathway** | **β** | **SE** | **95% CI** | **P** |
| --- | --- | --- | --- | --- |
| **Abuse composite to NSSI** |  |  |  |  |
| Total effect | 0.394 | 0.049 | [0.298, 0.491] | < 0.001 |
| Total indirect | 0.131 | 0.026 | [0.079, 0.183] | < 0.001 |
| Abuse → ED → NSSI | 0.068 | 0.019 | [0.031, 0.105] | < 0.001 |
| Abuse → Depression → NSSI | 0.033 | 0.013 | [0.008, 0.059] | 0.010 |
| Abuse → ED → Depression → NSSI | 0.030 | 0.010 | [0.011, 0.049] | 0.002 |
| Direct effect (Abuse → NSSI) | 0.263 | 0.048 | [0.169, 0.358] | < 0.001 |
| **Neglect composite to NSSI** |  |  |  |  |
| Total effect | 0.316 | 0.051 | [0.216, 0.417] | < 0.001 |
| Total indirect | 0.220 | 0.032 | [0.158, 0.281] | < 0.001 |
| Neglect → ED → NSSI | 0.134 | 0.032 | [0.071, 0.196] | < 0.001 |
| Neglect → Depression → NSSI | 0.027 | 0.012 | [0.003, 0.052] | 0.027 |
| Neglect → ED → Depression → NSSI | 0.058 | 0.016 | [0.027, 0.090] | < 0.001 |
| Direct effect (Neglect → NSSI) | 0.097 | 0.058 | [−0.016, 0.210] | 0.094 |
| **Effects from SA to NSSI** |  |  |  |  |
| Total effect | −0.059 | 0.056 | [−0.169, 0.050] | 0.288 |
| Total indirect | −0.009 | 0.019 | [−0.045, 0.028] | 0.634 |
| SA → ED → NSSI | −0.013 | 0.012 | [−0.037, 0.011] | 0.302 |
| SA → Depression → NSSI | 0.009 | 0.009 | [−0.009, 0.027] | 0.305 |
| SA → ED → Depression → NSSI | −0.006 | 0.005 | [−0.016, 0.004] | 0.298 |
| Direct effect (SA → NSSI) | −0.050 | 0.049 | [−0.147, 0.046] | 0.307 |

**Note.** The abuse composite combined emotional abuse and physical abuse; the neglect composite combined emotional neglect and physical neglect; sexual abuse (SA) was retained as a separate predictor. Standardized coefficients (STDYX) are reported; 95% CIs are MLR-based robust confidence intervals (delta method). Model fit: χ²(8) = 14.490, p = .070; CFI = 0.994; TLI = 0.974; RMSEA = 0.044 (90% CI = 0.000–0.079); SRMR = 0.013.

**Supplementary Table S6.** Standardized specific indirect effects with Benjamini-Hochberg FDR correction (MLR estimation)

| **Pathway** | **β** | **SE** | **95% CI** | **P** | **q (BH-FDR)** |
| --- | --- | --- | --- | --- | --- |
| EN → ED → NSSI | 0.097 | 0.024 | [0.049, 0.145] | < 0.001 | 0.003 |
| EN → ED → Depression → NSSI | 0.041 | 0.011 | [0.018, 0.063] | < 0.001 | 0.003 |
| PN → ED → NSSI | 0.091 | 0.022 | [0.047, 0.135] | < 0.001 | 0.003 |
| EA → ED → NSSI | 0.087 | 0.022 | [0.043, 0.131] | < 0.001 | 0.003 |
| PN → ED → Depression → NSSI | 0.038 | 0.012 | [0.015, 0.061] | 0.001 | 0.003 |
| EA → ED → Depression → NSSI | 0.036 | 0.011 | [0.015, 0.058] | 0.001 | 0.003 |
| EN → Depression → NSSI | 0.034 | 0.013 | [0.008, 0.059] | 0.009 | 0.019 |
| EA → Depression → NSSI | 0.026 | 0.011 | [0.004, 0.047] | 0.021 | 0.039 |
| PA → Depression → NSSI | 0.016 | 0.009 | [−0.002, 0.034] | 0.088 | 0.147 |
| PA → ED → NSSI | −0.016 | 0.013 | [−0.042, 0.010] | 0.223 | 0.316 |
| PA → ED → Depression → NSSI | −0.007 | 0.006 | [−0.018, 0.004] | 0.236 | 0.316 |
| SA → Depression → NSSI | 0.010 | 0.009 | [−0.008, 0.027] | 0.271 | 0.316 |
| SA → ED → Depression → NSSI | −0.005 | 0.005 | [−0.014, 0.004] | 0.293 | 0.316 |
| SA → ED → NSSI | −0.012 | 0.011 | [−0.034, 0.010] | 0.295 | 0.316 |
| PN → Depression → NSSI | −0.006 | 0.009 | [−0.023, 0.011] | 0.458 | 0.458 |

**Note.** Standardized coefficients (STDYX) are reported. 95% CI = MLR-based robust confidence interval (delta method). Benjamini–Hochberg FDR correction was applied to all 15 prespecified specific indirect effects (5 maltreatment types × 3 pathways). For paths with raw P < 0.001 in Mplus output, p was conservatively set to 0.001 for FDR computation; exact minimum q = .0025, reported as .003. BH-FDR q values are monotonized, so identical q values (e.g., .316) can occur for adjacent non-significant pathways.

**Supplementary Table S7.** Formal pairwise contrasts of total indirect effects (unstandardized, MLR estimation)

| **Contrast** | **Δ estimate** | **SE** | **P** | **95% CI** |
| --- | --- | --- | --- | --- |
| EN vs. EA | −0.002 | 0.012 | 0.882 | [−0.025, 0.022] |
| EN vs. PN | −0.007 | 0.015 | 0.648 | [−0.036, 0.022] |
| EN vs. PA | 0.074 | 0.021 | < 0.001 | [0.034, 0.115] |
| EN vs. SA | 0.079 | 0.029 | 0.006 | [0.022, 0.136] |
| EA vs. PN | −0.005 | 0.015 | 0.748 | [−0.035, 0.025] |
| EA vs. PA | 0.076 | 0.022 | 0.001 | [0.032, 0.120] |
| EA vs. SA | 0.081 | 0.033 | 0.013 | [0.017, 0.145] |
| PN vs. PA | 0.081 | 0.025 | 0.001 | [0.033, 0.129] |
| PN vs. SA | 0.086 | 0.033 | 0.009 | [0.021, 0.150] |
| PA vs. SA | 0.005 | 0.030 | 0.864 | [−0.053, 0.064] |

**Note.** Total indirect effects include all indirect pathways via emotion dysregulation, via depressive symptoms, and via the serial ED-to-depression pathway. Δ estimate = difference in unstandardized total indirect effects (e.g., EN vs. EA = EN-EA). All contrasts were tested using the MODEL CONSTRAINT command in Mplus.

**Supplementary Table S8.** Comparison of Model Fit Indices Among the Hypothesized and Alternative Models

| **Model** | **χ²(df)** | **CFI** | **TLI** | **RMSEA (90% CI)** | **SRMR** | **AIC** | **BIC** |
| --- | --- | --- | --- | --- | --- | --- | --- |
| Hypothesized (ED → Depression) | 14.923(10) | 0.995 | 0.981 | 0.034 (0.000–0.068) | 0.013 | 7163.164 | 7325.341 |
| Reversed-order (Depression → ED) | 14.923(10) | 0.995 | 0.981 | 0.034 (0.000–0.068) | 0.013 | 7163.164 | 7325.341 |
| Correlated-mediator (ED ↔ Depression) | 14.923(10) | 0.995 | 0.981 | 0.034 (0.000–0.068) | 0.013 | 7163.164 | 7325.341 |
| Parallel mediation (no ED→Depression) | 144.159(11) | 0.877 | 0.532 | 0.169 (0.145–0.194) | 0.051 | 7304.571 | 7462.694 |

**Note.** The parallel mediation model showed substantially poorer fit relative to the hypothesized model: ΔAIC = 141.407, ΔBIC = 137.353.

# 2 Supplementary **Appendix**

**Supplementary Appendix.** Mplus Syntax for the Primary Model (MLR Estimation)

The complete Mplus input syntax for the primary model is provided below.

TITLE: five maltreatment types on NSSI via ED and DS serial mediation (MLR);

DATA:

FILE = nssi.txt;

VARIABLE:

NAMES = en pn ea pa sa ders depression fsam1 fsam2 sex residence income age;

USEVARIABLES = en pn ea pa sa ders depression fsam1 fsam2 sex residence income age;

ANALYSIS:

ESTIMATOR = MLR;

MODEL:

NSSI BY fsam1@1

fsam2;

DERS ON en(a1)

pn(a2)

ea(a3)

pa(a4)

sa(a5);

DEPRESSION ON en(c1)

pn(c2)

ea(c3)

pa(c4)

sa(c5);

NSSI ON ders(b1)

depression(d1);

DEPRESSION ON ders(e1);

NSSI ON en(f1)

pn(f2)

ea(f3)

pa(f4)

sa(f5);

DERS ON sex residence income age;

DEPRESSION ON sex residence income age;

NSSI ON sex residence income age;

MODEL INDIRECT:

NSSI IND EN;

NSSI IND PN;

NSSI IND EA;

NSSI IND PA;

NSSI IND SA;

MODEL CONSTRAINT:

NEW(ind_en_ed ind_pn_ed ind_ea_ed ind_pa_ed ind_sa_ed

ind_en_ds ind_pn_ds ind_ea_ds ind_pa_ds ind_sa_ds

ind_en_eds ind_pn_eds ind_ea_eds ind_pa_eds ind_sa_eds

tot_en tot_pn tot_ea tot_pa tot_sa

diff_tot_en_ea diff_tot_en_pn diff_tot_en_pa diff_tot_en_sa

diff_tot_ea_pn diff_tot_ea_pa diff_tot_ea_sa

diff_tot_pn_pa diff_tot_pn_sa diff_tot_pa_sa);

ind_en_ed = a1*b1;

ind_pn_ed = a2*b1;

ind_ea_ed = a3*b1;

ind_pa_ed = a4*b1;

ind_sa_ed = a5*b1;

ind_en_ds = c1*d1;

ind_pn_ds = c2*d1;

ind_ea_ds = c3*d1;

ind_pa_ds = c4*d1;

ind_sa_ds = c5*d1;

ind_en_eds = a1*e1*d1;

ind_pn_eds = a2*e1*d1;

ind_ea_eds = a3*e1*d1;

ind_pa_eds = a4*e1*d1;

ind_sa_eds = a5*e1*d1;

tot_en = ind_en_ed + ind_en_ds + ind_en_eds;

tot_pn = ind_pn_ed + ind_pn_ds + ind_pn_eds;

tot_ea = ind_ea_ed + ind_ea_ds + ind_ea_eds;

tot_pa = ind_pa_ed + ind_pa_ds + ind_pa_eds;

tot_sa = ind_sa_ed + ind_sa_ds + ind_sa_eds;

diff_tot_en_ea = tot_en - tot_ea;

diff_tot_en_pn = tot_en - tot_pn;

diff_tot_en_pa = tot_en - tot_pa;

diff_tot_en_sa = tot_en - tot_sa;

diff_tot_ea_pn = tot_ea - tot_pn;

diff_tot_ea_pa = tot_ea - tot_pa;

diff_tot_ea_sa = tot_ea - tot_sa;

diff_tot_pn_pa = tot_pn - tot_pa;

diff_tot_pn_sa = tot_pn - tot_sa;

diff_tot_pa_sa = tot_pa - tot_sa;

OUTPUT:

STANDARDIZED;

STDYX;

CINTERVAL;

SAMPSTAT;

TECH1;

TECH4;
